# Mesenchymal Stem Cells and Their Exocytotic Vesicles

**DOI:** 10.3390/ijms24032085

**Published:** 2023-01-20

**Authors:** Hao Cai, Haidong Guo

**Affiliations:** 1 Academy of Integrative Medicine, Shanghai University of Traditional Chinese Medicine, Shanghai 201203, China; 2 Department of Anatomy, School of Basic Medicine, Shanghai University of Traditional Chinese Medicine, Shanghai 201203, China

**Keywords:** mesenchymal stem cells, differentiation, extracellular vesicles, immunomodulation, cardiocerebral diseases, signaling pathways

## Abstract

Mesenchymal stem cells (MSCs), as a kind of pluripotent stem cells, have attracted much attention in orthopedic diseases, geriatric diseases, metabolic diseases, and sports functions due to their osteogenic potential, chondrogenic differentiation ability, and adipocyte differentiation. Anti-inflammation, anti-fibrosis, angiogenesis promotion, neurogenesis, immune regulation, and secreted growth factors, proteases, hormones, cytokines, and chemokines of MSCs have been widely studied in liver and kidney diseases, cardiovascular and cerebrovascular diseases. In recent years, many studies have shown that the extracellular vesicles of MSCs have similar functions to MSCs transplantation in all the above aspects. Here we review the research progress of MSCs and their exocrine vesicles in recent years.

## 1. Mesenchymal Stem Cells

As a pluripotent stem cell capable of self-renewal and differentiation into multi-lineage cells, mesenchymal stem cells (MSCs) can be obtained from a variety of human tissues and organs such as bone marrow, bone trabeculae, adipose tissue, brain, lung, pancreas, synovial fusion, synovium, peripheral blood [1,2,3,4,5,6,7,8].

Most MSCs express MSC-related surface markers CD44, CD73, CD90, CD105, and some express CD29, CD49e, CD54, CD106, CD146, CD166, CD271, SSEA4, Notch-1, HLA-ABC, Stro1 [9]. Expression of different markers implies different germline origins, tissue origins, and functional characteristics. Different origins imply different differentiation abilities, clinical benefits, and cultural characteristics [10].

Therefore, different types of MSCs have different applications in transplantation and co-culture. For example, bone marrow mesenchymal stem cells (BMSCs) are abundant in bone tissue, and co-culture of BMSCs with cartilage tissue increased the proliferation rate of chondrocytes [11], reduced chondrocyte apoptosis and inflammation [12]. Co-culture of BMSCs with estrogen receptor-positive (ER+) breast cancer cells increased resistance to standard anti-estrogen drugs [13] and enhanced osteoclastogenesis [14]. Adipose-derived mesenchymal stem cells (ADMSCs) regulated cell proliferation, improved fibroblast migration, and promoted capillary structure formation [15]. Pancreatic-derived mesenchymal stem cell (PMSCs) implantation rescued damaged exocrine pancreas and islet β-cells [16]. Human umbilical cord-derived mesenchymal stem cells (hUC-MSCs) contributed to injured neuronal repair and motor recovery [17]. In recent years many research teams have investigated the application of MSCs in various aspects. Here we review the progress of research on MSCs and their exocytotic vesicles.

## 2. Osteogenic Differentiation and Repair

Osteoblastogenesis needs three stages: proliferation, matrix maturation, and mineralization [18]. This differentiation process depends on transcription factors Runx2 and Osx [19,20,21,22]. Osteoblasts can then develop into osteocytes that are encapsulated by mineralized bone, have mechanosensory and metabolic functions, and regulate bone remodeling [23,24,25,26]. Among the many molecules affecting osteogenic differentiation, TGF-β family members, bone morphogenetic proteins (BMPs), TGF-βs, activins, and inhibins played an important role in the early stages of differentiation of BMSCs into mature stromal secretory osteoblasts and osteocytes [27]. TGF-β ligands regulated osteoblast differentiation through Smad2 and Smad3 signaling pathways [28,29,30]. BMPs signal through BMPRII and ALK-2, BMPRIA, or BMPRIB to promote osteoblast differentiation in the form of increased alkaline phosphatase expression and activity, osteocalcin, and bone bridging protein expression [31,32,33,34,35,36]. BMP ligands promoted osteogenic differentiation via Smad, extracellular signal-regulated kinase (Erk) 1/2 mitogen-activated protein kinase (MAPK), p38 MAPK, and JNK-related pathways. TGF-βs promoted the proliferation and early differentiation of MSCs to osteoblasts and chondrocytes but inhibited late transformation from maturing osteoblasts and chondrocytes. BMP-2 promoted the selection and differentiation of chondrogenic and osteogenic lineages and also promoted late osteoblast differentiation and matrix mineralization [27]. The osteogenic potential of BMSCs was affected by environmental conditions, such as low oxygen conditions to maintain osteogenic potential and high oxygen to interfere with the metabolic chromatin osteogenic axis and reduce citrate carrier (CiC) activity, leading to osteogenic defects [37]. Activation of Wnt signaling promoted osteogenic differentiation of BMSCs [38]. Wnt3α stimulates osteogenic differentiation by activating TAZ through pp1a-mediated dephosphorylation [39]. Yes-associated protein (YAP)/TAZ mediated Wnt signaling-induced osteogenesis [40]. DHA-rich phosphatidylcholine (DHA-PC) upregulated the expression of the Wnt/β-catenin pathway and osteogenic transcription factors in BMSCs and promoted osteogenic differentiation [41]. circStag1 is a key osteoporosis-related circRNA that interacts with human antigen R (HuR), an RNA-binding protein, and promotes the translocation of HuR to the cytoplasm. Increasing HuR levels in cells stimulated osteogenesis in BMSCs by stabilizing and enhancing the expression of low-density lipoprotein receptor-related protein 5/6 (Lrp5/6) and β-catenin, thereby activating the Wnt signaling pathway [42]. Melatonin-mediated MEK1/2 and MEK5 affected bone mechanical properties (i.e., ultimate stress), bone density, and microarchitecture (i.e., trabecular number, separation, and junction density) by increasing the expression of the osteogenic genes RUNX2, BMP-2, FRA-1, and OPG and decreasing the expression of peroxisome proliferation-activated receptor γ (PPARγ) in MSCs [43]. In addition, triboelectric stimulation and magneto-mechanical stress also promoted osteogenesis in BMSCs [44,45]. Electrical field stimulation promoted osteogenic differentiation by upregulating Runx2/OCN gene expression with the BMP/Smad4 pathway. Mechanical stimulation activated the typical Wnt/β-linked protein signaling pathway with significantly elevated ALP activity and Sp7 gene expression [46]. Negative pressure (NP)-treated MSCs induced osteogenesis by activating macroautophagy/autophagy through AMPK-ULK1 signaling [47]. In addition to BMSCs, adipose-derived mesenchymal stem cells (ADMSCs) also possess osteogenic potential, and inhibition of miR-150–15p promoted osteogenesis in ADMSCs by regulating Notch3 upregulation of pFAK, pERK1/2, and RhoA expression [48].

BMSCs also possess the ability to differentiate into mature chondrocytes [49]. Magnetic field and TGF-β stimulation both significantly enhanced the efficiency of chondrogenic differentiation of BMSCs [50,51]. Upregulation of PLCE1, CaMKII-β, and downstream NFATc1 expression activated Wnt signaling and enhanced the expression of chondrogenic markers such as SOX9, which enhanced chondrogenesis in MSCs [52]. The nuclear factor-kappaB (NF-κB) family plays an important role in biological processes such as mechanical processes, immunity, inflammation, and oxidative stress that can be activated by chemokines, pro-inflammatory, and degradation factors [53]. The deficiency of NF-κB member RelA during the culture of BMSCs increases chondrogenic differentiation [54]. Co-culture with cartilage tissue increased the proliferation rate of chondrocytes [11], decreased the activation levels of MAPK, Erk 1/2, and p38 in chondrocytes [55], increased the levels of extracellular matrix (ECM) proteins Col-II and SOX9 through upregulation of mediators of the autophagic phosphatidylinositol 3 kinase/AKT/mammalian target of rapamycin (mTOR) pathway, and reduced chondrocyte apoptosis and inflammation. BMSCs not only have good osteogenic ability but also promote osteoclastogenesis under certain conditions. Co-culture of BMSCs with breast cancer cells enhanced osteoclastogenic ability [14].

Bioengineering techniques are now widely used to assist stem cell osteogenesis. The protein biomineralized hydroxyapatite coating of Apt19s coating on the titanium surface via oxidized hyaluronic acid (OHA) enhanced BMSCs migration with sustained release of Apt19s, promoted BMSCs recruitment at the peri-implant interface and improved osteogenic differentiation [56]. Hydrogen-terminated nanocrystalline diamond (NCD) provided better adhesion and proliferation than conventional materials such as titanium. Fibroblasts grow worst on hydrogenated NCD, which can be used to improve bone fixation and osseointegration [57]. Increasing levels of reactive oxygen species (ROS) in the bone microenvironment are a hallmark of osteoporosis and lead to the dysfunction of MSCs, resulting in their senescence and severely compromising their osteogenic potential [58]. MSCs with a lipoic acid-containing extracellular antioxidant protective layer are effectively protected from the degradation of reactive oxygen species and improved survival and engraftment efficiency [59]. Bilayer hydrogels loaded with two types of peptides induced macrophage M1-type polarization at the appropriate time to activate the immune system to kill bacteria or modulate macrophage M2-type polarization to promote the formation of an anti-inflammatory microenvironment, thereby promoting bone regeneration associated with MSCs [60]. Synthetic fibrinopeptide hydrogels (GR) self-assembled from β-sheet RADA16-grafted glucomannan promoted proliferation and osteogenic differentiation of BMSCs and further enhanced this function by inducing macrophage M2 polarization for effective M2 macrophage BMSCs crosstalk [61]. Hydrogel flow-induced mechanical stimulation enhanced the quality of minerals deposited by MSCs during the culture of 3D bioprinted bone tissue constructs [62]. Calcium phosphate (CaP) bioceramic possessed good osteoinductive and biodegradable properties, and co-culture with BMSCs promoted their proliferation and osteogenic capacity [63]. Polyvinyl alcohol (PVA) hydrogel composite film with a cellulose fiber skeleton of delignified wood (named white wood, WW) combined with curcumin (Cur) and phytic acid (PA) effectively exerted antibacterial, anti-inflammatory, and osteogenic effects and promoted adhesion, proliferation, and osteogenic differentiation of BMSCs [64]. In some skeletal external devices, the adhesion of bone to the device that accompanies bone regeneration increases the difficulty of device removal and affects the microenvironment of bone healing. Combining poly (ethylene glycol) methyl ether methacrylate (poly (PEGMA)) with optimal PEG chain length on the tenting screw technology (TST) titanium surface reduced osteogenic integration on the implant surface by preventing cellular adhesion integration, thus reducing the difficulty of implant removal [65]. MSCs significantly upregulated chondrogenesis-related genes on magnetic scaffolds with citric acid-coated magnetic nanoparticles (MNPs-CAG) and osteogenesis-related genes on magnetic scaffolds with polyvinylpyrrolidone-coated MNPs [66]. Bioprinted scaffolds consisting of 8% methacrylamide gelatin (GelMA)/1% methacrylamide hyaluronic acid (HAMA) as an encapsulation system for rat bone marrow-derived macrophages (BMM) and 3% alginate/0.5 mg/mL graphene oxide (GO) as an encapsulant for rat BMSCs improved the inflammatory microenvironment and further accelerates bone repair [67]. The exploration of many metallic, nonmetallic, gel, and nanoparticle materials offers good prospects for the clinical application of MSCs.

## 3. Adipose Differentiation and Regulation

The current study shows that the differentiation of MSCs to adipocytes and to osteoblasts is transcriptionally regulated by two key transcription factors, PPARγ and Runx2. PPARγ1 is highly expressed during adipocyte differentiation and regulates the expression of adipogenesis-related genes [68]. eCCAAT/enhancer binding proteins (C/EBPs) β and δ (encoded by Cebpb and Cebpd, respectively) induce differentiation of MSCs to adipocytes, C/EBPβ is phosphorylated by Erk, MAPK, and glycogen synthase kinase 3β (GSK3β) [69,70], which induce C/EBPα and PPARγ (encoded by Cebpa and Pparg, respectively) [71,72], and C/EBPα and PPARγ jointly and drive differentiation toward adipocytes [72]. C/EBPβ is downregulated at late stages of differentiation, while PPARγ inhibits chondrogenesis [73,74,75,76]. Runx2 induced osteogenic gene expression, thereby increasing osteoblast differentiation [77]. Runx2 promoted differentiation of MSCs to preosteoblasts and related gene expression in the early stages while inhibiting differentiation to adipocytes [78], followed by increased alkaline phosphatase activity and mineralization driven by expression of the transcription factor osteosteroid (Osx, encoded by Sp7) [22]. Runx2 decreased in the late stages of osteogenic differentiation [79]. sLZIP prevented PPARγ2 target gene expression and adipocyte differentiation, upregulated Runx2 transcriptional activity by inhibiting PPARγ2 activity and promoted osteoblast differentiation without affecting chondrogenesis and osteoclastogenesis [80].

Activation of the Hedgehog signaling pathway blocked lipogenic differentiation by inhibiting PPARγ and C/EBPα expression and lipid accumulation in 3T3-L1 and C3H10T1/2 cells [81]. Fibroblast growth factor (FGF) 2, FGF4, FGF, and FGF8 upregulated Runx2 expression and promoted alkaline phosphatase activity to facilitate mineralization during late osteogenic differentiation [81,82]. FGF1, FGF2, and FGF2 played a dual role in regulating lipogenesis and osteogenic differentiation [83]. Among BMP ligands, BMP-3b inhibited osteogenic differentiation by binding to ActRIB/ALK-4 and ActRIIB and subsequently activating Smad3, thereby affecting lipogenic differentiation [84]. BMP-4 had a pro-adipogenic effect and directed pluripotent C3H10T1/2 cells to the adipocyte lineage and promoted white adipose tissue differentiation [85,86]. BMP-7 induced human mesenchymal stem cells to form a lipogenic lineage in high-density micro-mass culture, and the BMP-7-induced Smad and p38 MAPK pathways with nuclear coactivator PGC1 (PPARγ coactivator-1) played a role in thermogenic regulation. They promoted brown adipose tissue differentiation [87,88]. TGF-βs promoted the proliferation and early differentiation of MSCs to adipocyte progenitors and inhibited later conversion to mature adipocytes [27]. Snail1 plays a key role in TGF-β1-induced maintenance of stemness in MSCs [89]. Myogenesis inhibitor is a key negative regulator of skeletal mass, promotes adipogenesis, and inhibits myogenesis in C3H10T1/2 pluripotent MSCs [90,91]. Blocking the Notch signaling pathway promoted autophagy-mediated lipogenic differentiation of MSCs via PTEN-PI3K/AKT/mTOR pathway [92]. Inhibition of nicotinamide adenine dinucleotide (NAD+) biosynthesis during the differentiation of MSCs to adipocytes effectively promoted the adipogenic transcriptional program [93]. Human circadian genes regulated the differentiation of MSCs and thus influenced human metabolism. MSCs were central to subcutaneous adipose tissue, visceral adipose tissue, and skeletal muscle tissue in circadian regulation and extracellular matrix remodeling [94]. Thus, MSCs have great potential in the study and application of the human aging process and many chronic and geriatric diseases.

### 3.1. Liver, Kidney, and Pancreas

MSCs released a variety of trophic and regulatory molecules, including growth factors, proteases, hormones, cytokines, and chemokines [95], which regulate inflammatory, fibrotic, and hypoxic responses by releasing these soluble factors in close interaction with the local microenvironment as a way to promote tissue repair and regeneration [96]. In addition, anti-apoptotic secreted and mitogenic growth factors, such as hepatocyte growth factor (HGF), insulin-like growth factor (IGF-1), vascular endothelial growth factor (VEGF), FGF2, basic nerve growth factor, epidermal growth factor (EGF), and platelet-derived growth factor (PDGF), contributed to cellular proliferation at the site of injury. BMSCs also released angiogenic (e.g., VEGF, angiopoietin-1, and monocyte chemotactic protein (MCP)-1) and chemokines, which further aid in the recovery of damaged tissues by promoting angiogenesis and angiogenesis [97]. These factors also mediated ECM remodeling by secreting ECM and cell adhesion proteins and regulated the activity of matrix-degrading mitochondrial membrane potentials (MMPs) [98].

MSCs also exerted anti-fibrotic and cytoprotective effects through immunomodulation, inhibition of TGF-β1 activity, suppression of oxidative stress, and stimulation of matrix remodeling by promoting MMP-induced collagen degradation and/or downregulation of tissue matrix metalloproteinase inhibitor (TIMPs). For example, transplantation of BMSCs decreased TGF-β expression and intracellular Smad2 downstream phosphorylation (TGF-β1 binding to its receptor), which in turn inhibited the number of α-SMA-positive cells [99,100,101], resulting in lower levels of myofibroblast proliferation and differentiation, reduced myofibroblast-induced ECM production and epithelial-mesenchymal transition (EMT), and ultimately inhibited the transition from tubular epithelium to myofibroblasts phenotypically, eventually altered renal fibrosis process [101]. TGF-β1 inhibition of BMSCs may be related to the release of HGF and TNF-stimulated gene 6 (TSG-6) from the cells [98,102]. In hypertensive chronic kidney disease, BMSCs could exert their antihypertensive effects by inhibiting NLRP3 inflammatory vesicle assembly and activity, suppressing immune cell infiltration such as macrophages and T cells, inhibiting the prohypertensive component of RAAS, and suppressing sympathetic activation [103,104]. In a renal vascular hypertension model, BMSCs attenuated the expression of intrarenal RAS components, including intrarenal angiotensinogen, renin, ACE, and AT1R, to prevent elevated systolic blood pressure (SBP) [105]. BMSCs inhibit macrophage and T cell-induced unilateral ureteral obstruction (UUO) and hypertension [103,106,107]. MSCs secreted anti-apoptotic (IL-6, IGFBP-2) and anti-inflammatory (IL-1Ra) cytokines to inhibit hepatic stellate cell activation and exerted anti-fibrotic effects in liver fibrosis [108]. Pancreatic-derived mesenchymal stem cells (PMSCs) implanted in the renal capsule of streptozotocin (STZ)-induced diabetic mice were able to regenerate the exocrine pancreas, enhance IL-6 activation of tumor necrosis factor-α (TNF-α) and interferon-γ (IFN-γ) inhibition of IL-17, and rescue damaged exocrine pancreas and islet β-cells [16].

The role of MSCs in regulating metabolism and the metabolic environment is also mostly related to their cytoprotective effects. For example, MSCs improved insulin sensitivity in high-fat diet and streptozotocin (STZ)-induced T2D and leptin receptor-deficient (db/db) mice, reduced β-cell mortality, and decreased inflammation and fat content in adipose tissue and liver [109]. In type 2 diabetes (T2D), MCP-1 prevented the conversion of BMSCs into adipocytes, increased bone cell density, and improved metabolism through its antagonistic effects [110]. Thus, the regulation of MSCs by genetic engineering techniques has a practical future. For example, circARHAP12-mediated autophagy inhibited high-glucose-induced apoptosis in MSCs. CircARHAP12 directly interacted with miR-301b-3p to regulate the expression of miR-301b-3p target genes ATG16L1 and ULK2 and downstream signaling pathways to promote the therapeutic role of MSCs in diabetic wound healing [111]. Human alpha-1 antitrypsin (hAAT)-engineered mesenchymal stromal cells (hAAT-MSCs) inhibited macrophage migration and shifted M1-like phenotype to M2 phenotype in vitro and in vivo to improve co-transplanted islet/beta cell survival [112].

### 3.2. Cardiovascular

Numerous studies have demonstrated that MSCs transplantation can effectively limit infarct size after myocardial infarction and that mobilization, homing, migration, adhesion, and preservation of MSCs play a significant therapeutic role in cardiovascular diseases such as infarction, ischemia-reperfusion, and heart failure. MSCs played an important role in protecting cardiomyocyte survival, promoting the proliferation of vascular endothelial cells and lymphocytes, and promoting vascular neogenesis. Differences in the angiogenic biological activity of MSCs are also associated with the expression of several genes involved in angiogenesis, such as HGF, angiopoietin-1 (ANGPT), or matricellular proteins (fibronectin (FN), insulin-like growth factor binding protein 7 (IGFBP7)) [113]. In addition, stromal cell-derived factor-1 (SDF-1) and stem cell factor (SCF) contributed to reducing infarct size and collagen content and protecting the heart [114]. MSCs secreted chemokine (C-C motif) ligand 7 (CCL7)/chemokine (C-C motif) receptor 1 (CCR1) to activate downstream CBP/P300 acetylation of KLF5 to promote CXC ligand 5 (CXCL5) transcription and secreted TGF-β to reverse the effect of KLF5 on transcriptional activation by regulating SMAD4, and CXCL5 promoted distant tumor metastasis and angiogenesis [115]. ADMSCs transplantation promotes diabetic skin wound healing by improving fibroblast migration capacity and promoting capillary structure formation [15]. BMSCs promoted angiogenesis by upregulating the endothelial transcription factor forkhead box protein C2 (FOXC2) and its downstream gene αvβ3 integrin/CD61 in B-MSCs [116].

The products of MSCs contain various pro-angiogenic factors [117,118], such as VEGF [119,120,121], PDGF [122], angiopoietin [123], HGF [119], metalloproteinases [124], and microRNAs such as miR-21 [119,125], miR-125a [126], and miR-377 [127], all of which have enhanced cell survival and promote angiogenesis. Thus, they played an important role in the preservation and recovery of cardiac function because cardiomyocytes are dependent on oxygen supply from the blood and are very sensitive to reduced blood perfusion [128,129]. In the sudden blockage of coronary arteries (acute infarction), cardiomyocytes died within minutes. Capillary thinning is, therefore, a sign of ventricular insufficiency [130,131]. An increase in pro-angiogenic factors has also been shown to promote recovery of cardiac function [132,133].

MSCs affected cardioprotective by affecting oxidative stress and modulating immunity. Sod-adenovirus-infected BMSCs maintained high SOD3 expression and delivered these BMSCs to the infarcted myocardium of mice, resulting in reduced oxidative stress and improved cardiac function [134]. The antioxidant effect may be associated with increased expression of superoxide dismutase (SOD). In an in vitro model of ischemia-reperfusion injury in ventricular cardiomyocytes, BMSCs were shown to secrete SOD3 and reduce ROS levels [135]. Post-infarction infusion of MSCs reduced the proportion of proinflammatory M1 macrophages in the infarcted myocardium while driving monocytes to alternatively activated M2 state polarization [136]. This reduced the amount of pro-inflammatory cytokines (including TNF-α and IL-1β) in the infarcted myocardium. The presence of M2 macrophages further produced soluble factors, including TGF-β, IL-10, HGF, PGE2, and IDO, to suppress the pro-inflammatory state [137]. In a mouse model of myocardial infarction, MSCs injection significantly reduced the M1 phenotype of macrophages, decreased the expression of interleukin-1β (IL-1β) and IL-6, increased the expression of IL-10, and increased alternate monocyte/macrophage activation. MSCs could repair the myocardium through IL-10-mediated infiltration of pro-inflammatory macrophages into anti-inflammatory macrophages at the infarct site [119,136]. In a porcine heart failure combined with a myocardial infarction model, viability was enhanced, and vascular regeneration was promoted by activating regulatory T cells (Tregs) and reducing inflammatory cells [138]. MSCs expressed Fas ligand (FasL), directly triggered t-cell apoptosis via the Fas/FasL pathway, and promoted inhibition of T-cell proliferation via the programmed death 1 pathway [139,140]. Combined MSCs and MSC-Exos injections can obtain more effective anti-inflammatory and vascular tissue repair by reducing the expression of inflammatory factors such as IL-6 and TNF-α and increasing the expression of recombinant SDF-1 [141]. MSCs administered intravenously to patients with chronic heart failure are involved in regulating adaptive immunity and myocardial remodeling by secreting HGF, inhibiting T cell proliferation, and reducing the proliferation of Th 1, Th 2, and cytotoxic T cells [142]. iPSC-MSCs reduced inflammation by decreasing serum levels of inflammatory cytokines such as TNF-α and IL-6, thereby treating atherosclerosis [143]. CD73 converted adenosine monophosphate (AMP) to adenosine, thereby inhibiting t-cell activation. In a MIRI rat model, MSCs injected into damaged myocardium were found to mediate CD73 activity and attenuate the infiltration of innate immune cells, thereby protecting cardiac function [144]. In vitro, BMSCs had a potent immunosuppressive capacity and inhibited lymphocyte proliferation and antibody production. Plasma cells from patients with end-stage heart failure have a high IgG3 output. In vitro experiments have shown that bone marrow stem cells can inhibit IgG3 production, thereby preventing ventricular remodeling and slowing the progression of heart failure after myocardial infarction [145]. CD4 is normally expressed on the surface of human T cells, and the Th1 in CD4 cells plays a role in humoral immunity [146]. CD4/CD8 was an important indicator of immune regulation [147]. Co-transplantation of BMSCs with pluripotent stem cell-derived cardiomyocytes into mice revealed that BMSCs directly affect activated lymphocytes through intercellular contacts, thereby decreasing the CD4/CD8 ratio and the proportion of th1-positive cells among CD4-positive cells, and decreasing the secretion of various inflammation-associated cytokines. In addition, this pathway increases the number of cardiomyocytes and enhances myocardial contraction [148]. Immune factors IFN-γ and IL-1β influenced MSCs to reverse Nor1-dependent iPS-CM hypertrophy via VEGF [149]. Patients with chronic heart failure injected with BMSCs showed a significant decrease in CD4-positive cells and NK cells and a significant improvement in left ventricular end-systolic volume (LVESV) and LVEF [150].

The immunomodulatory and angiogenic effects of MSCs can be more effectively exploited in combination with bioengineering. For example, protein nanoparticles combined with encapsulated MSCs hydrogels supported the proliferation, migration, and angiogenesis of MSCs in vitro. The hydrogels affected ventricular remodeling after myocardial infarction by slow releasing of immunomodulatory factors [151]. The angiogenic effect was enhanced by genetic alterations inducing MSCs to overexpress certain proteins (e.g., CXCR-4, GATA-4, Akt, SDF-1a) or pharmacological modulation of MSCs (e.g., angiotensin II, diazoxide) [152,153,154,155,156,157].

Although MSCs have limited differentiation ability, some studies have proved that MSCs have the ability to differentiate into beating myocardial cells in vitro [158]. By inducing non-specific DNA demethylation, cells with increased spontaneous beating, myocardial cell-like structure, protein expression, action potential, and connexin 43 markers have been obtained, and it has been proved that MSCs increase the overall expression of connexin 43 in infarcted myocardium in mouse models [159]. The differentiation of MSCs into cardiomyocytes has received less attention in recent years. On the one hand, the differentiation efficiency of MSCs is lower than that of human induced pluripotent stem cells; On the other hand, the differentiation ability of MSCs is often affected by the extracellular matrix. For example, under the influence of laminin, BMSCs will have an increase in adipogenesis markers, while ADMSCs will have an increase in neurogenesis markers. MSCs transplantation often faces a severe cell survival environment [160]. In contrast, the study of paracrine function and transplantation survival efficiency of MSCs provides a broad prospect for the application of MSCs.

### 3.3. Neuro

MSCs effectively protect neuronal cells, reduce apoptosis, and promote neuroprotrusion growth, neurogenesis, synaptic transmission, and neurotransmitter transmission to promote recovery from neurological injury. In terms of anti-apoptosis, MSCs resisted astrocyte apoptosis by inhibiting endoplasmic reticulum (ER) stress, IL-6/STAT3 signaling pathway, and Cx43/Nrf2 interaction [161,162,163,164]. Expression of markers of pro-apoptotic processes such as Bax was downregulated, and expression of anti-apoptotic proteins such as Bcl-2 was upregulated after MSCs transplantation. Endoplasmic reticulum stress led to damage and cytotoxicity of folding proteins such as GRP78, XBP-1 PERK, eIF2a, ATF4, and CHOP, leading to apoptosis and inhibition of blocking endoplasmic reticulum stress-induced pro-apoptotic pathways appears to be more dominant than anti-apoptotic promotion [162,163]. Il-6, a key factor in astrocyte proliferation and the blood–brain barrier coherence, was upregulated after cerebral infarction. Astrocytes co-cultured with MSCs then significantly improved IL-6 secretion. Meanwhile, the anti-apoptotic mechanism of IL-6 in astrocytes may be directly involved in the IL-6/STAT3 signaling pathway. In addition, MSCs-based treatment enhanced the expression of connexin 43 (Cx43) and nuclear factor red lineage-related factor 2 (Nrf2). It promoted the antioxidant response of astrocytes, including increased secretion of HO-1 enzyme, and impaired apoptosis [161,165,166].

MSCs also promote injured nerve recovery by promoting neuroprotrusion growth, neurogenesis, synaptic transmission, and neurotransmitter delivery. For example, human pluripotent stem cell-derived external mesenchymal stromal cells (hPSC-EMSCs) are significantly enriched in secreted factors such as nerve growth factor (NGF), platelet-derived growth factor AA, and transforming growth factor-β, which are involved in neurogenesis, synaptic transmission, and neurotransmitter delivery, respectively. The mechanism is to enhance NGF-induced neuroprotrusion growth and neuronal differentiation of NPCs through the ERK/CREB pathway, reduce brain injury size, promote endogenous neurogenesis, reduce the inflammatory response, and improve functional recovery. hPSC-EMSCs had higher neuroprotective potential than human umbilical cord-derived MSCs (hUC-MSCs) through anti-apoptotic and neuroprotrusion growth as well as promote neurogenesis, which can better promote endogenous neurogenesis and attenuate astrocyte proliferation and microglia proliferation [167]. Transplantation of MSCs after traumatic brain injury (TBI) improved neurological deficits by modulating inflammation and enhancing neurotrophic factor expression [168]. Transplantation of human umbilical cord-derived mesenchymal stem cells (hUC-MSCs) promoted motor recovery from spinal cord injury (SCI) by promoting upregulation of GABAR subunits (β3 and γ2) and KCC2 in injured neurons via BDNF [17]. In addition, MSCs promoted cell survival by regulating autophagy. For example, the co-culture of BMSCs with Aβ-treated neuronal cells significantly increased the induction of autophagosomes, final maturation of late AVs, and fusion with lysosomes. And reduced the level of Aβ in the hippocampus, the survival of hippocampal neurons was increased, and upregulation of BECN1/Beclin 1 expression in the AD model significantly enhanced autolysosome formation and clearance of Aβ in the AD model, leading to increased survival of neurons against Aβ toxicity [169].

The neuroinflammatory response and increased blood–brain barrier permeability are vicious cycles in neurological injury, and inhibition of this cycle can effectively protect the nerves [162]. MSCs can exert anti-inflammatory effects by downregulating pro-inflammatory cytokines, preventing leukocyte penetration, and promoting the polarization of microglia to the M2 phenotype [170]. Human amniotic mesenchymal stem cell (hAMSC) injection significantly increased the integrity of the blood–brain barrier, reduced TNF-α and iNOS, and inhibited microglia conversion to the pro-inflammatory M1 phenotype [162,171]. ADMSCs significantly improved microvascular disruption, congestion, and distortion and reduced the blood–brain barrier leakage in stroke rats [170]. Membrane-linked protein A1 (ANXA1) is expressed in microglia and BMVECs and acts as an anti-inflammatory agent via formylated peptide receptors (FPRs) agonists [172,173,174]. ANXA1 induced microglia to phagocytose apoptotic cells and debris without eliciting a pro-inflammatory response and promoted microglia polarization and migration. Thus, the molecular mechanism by which MSCs preserve the blood–brain barrier was related to the stabilization of the endothelial layer via the ANXA1/FPR axis and anti-inflammatory effects [175]. MSC transplantation significantly downregulated MMP9 activity without affecting MMP2 activity [176]. Attenuating the upregulation of MMP-9 in extravasated neutrophils and resident cells contributes to the preservation of the blood–brain barrier and reduce infarct volume and neurological deficits after ischemic stroke. In contrast, elevated MMP-2 levels may promote endothelial cell proliferation and maturation, further improving neurological prognosis after stem cell administration [177,178]. In a transient middle cerebral artery occlusion (MCAO) model, MSC transplantation significantly reduced IgG infiltration by decreasing the expression of MMP-9, TNF-α and pro-inflammatory factors (IL-1β, IL-6) and neutrophil penetration [176].

### 3.4. Immunomodulation

As mutually antagonistic inflammatory factors in immune regulation, pro-inflammatory cytokines mainly include IL-1β, TNF-α, IL-6, IL-15, IL-17, IL-18, and anti-inflammatory factors mainly include TNF-stimulated gene 6, IL-4, IL-10, IL-13, IL-37 [179]. MSCs have good immunomodulatory properties, capable of immune interaction with major immune cells, including natural killer cells, macrophages, dendritic cells (DCs), T cells, and B cells, among other cells.

Macrophages can be polarized by different signals into the classical M1 phenotype or alternatively activated M2 subtype [180]. M1 macrophages exhibit potent antimicrobial properties and are responsible for promoting the Th1 response (characterized by increased production of pro-inflammatory cytokines, including IL-1β, IL-6, and TNF-α). In contrast, M2 macrophages secrete fewer pro-inflammatory cytokines (characterized by high levels of IL-10 production and low levels of IL-12, TNF-α, and IL-1α), promote th2-type immune responses, and display a hyperphagocytic phenotype [181]. MSCs polarized the proinflammatory M1 macrophages into M2 subtypes. M1 macrophages co-cultured with BMSCs for 72 h expressed CD206 (a marker of M2 macrophages), accompanied by high levels of anti-inflammatory cytokines IL-10 and low levels of proinflammatory cytokines, including IL-12, TNF-α and IL-1α [182]. The proportion of M2 macrophages was significantly reduced, and toll-like receptor 4 (TLR4) expression was significantly increased in BMSCs-treated mice, suggesting that BMSCs inhibited the shift of pro-inflammatory macrophages to the M2 subtype through the TRL4 signaling pathway [183]. The underlying mechanism may be that lipopolysaccharide (LPS) and TNF-α enriched in the inflammatory microenvironment activate TLR4 and TNF receptor 1 (TNFR1) on BMSCs, inducing the NF-κB signaling pathway, and upregulated cyclooxygenase (COX)-2 expression [184]. Yap proteins in the Hippo pathway can regulate the inflammatory response. MSCs regulated the inflammatory response by activating the MSCs control the assembly of NLRP3 inflammatory vesicles by activating the Hippo pathway and regulating the interaction between Yap and β-linked proteins [185]. The inflammatory chemokines CCL2 and CXCL2 secreted by BMSCs played an important role in polarizing mouse peritoneal macrophages to the IL-10 phenotype [186]. MSCs secretome contained tissue growth factor-β3 (TGF-β3), thrombospondin-1 (TSP-1), miR-182, miR-223, and miR-322, which contribute to the polarization toward anti-inflammatory macrophages and reducing the amounts of inflammatory cytokines such as TNF-α and IL-12 released from injured tissues [187,188].

MSCs inhibited B-cell proliferation in vitro and suppressed B-cell differentiation and structural expression of chemokine receptors [140,189,190]. MSCs from human-term placental amniotic membranes (hAMSCs) and the conditioned medium mesenchymal stem cells (CMhAMSCs) generated from their culture strongly inhibited the proliferation of CPG-activated B cells. Furthermore, CM-hAMSCs blocked B cell differentiation, resulting in an increased proportion of mature B cells and reduced the formation of antibody-secreting cells [191]. It was shown that hAMSCs have immunosuppressive effects on B cells and constitutively express high levels of the immunosuppressive ligand programmed cell death ligand 1 (PD-L1) in response to IFN-γ [192]. In mouse models, placental MSCs (P-MSCs) inhibited the proliferation and further differentiation of B cells [193]. LPS-stimulated B cells co-cultured with MSCs have shown that B cell proliferation and differentiation can be inhibited [194], and in vitro studies have shown that BMSCs support the survival, proliferation, and differentiation of normal human B cells into antibody-secreting cells [195,196]. It is possible that this is the result of different experimental conditions in the hypersensitive response of immune cells. However, the possibility that BMSCs contain different subtypes of MSCs cannot be excluded. Co-cultures of MSCs and lymphocytes mutually exerted an inhibitory effect, which inhibited the growth of MSCs and enhanced their immunosuppressive capacity [197]. Co-cultures of MSCs and lymphocytes showed that MSCs stimulate B-cell antibody secretion, and whether B-cell antibody secretion is inhibited or promoted is determined by the dose of MSCs [196].

MSCs were able to inhibit the proliferation, maturation, and differentiation of DCs, thus exerting immunomodulatory and immunosuppressive effects [198]. DCs initiated T cell immunomodulation and regulated T cell differentiation to Th1, Th2, Th17, or Treg subpopulations. DCs also indirectly regulate T cell subpopulation differentiation by activating regulatory cytokine production by intrinsic lymphocytes [199]. Immature dendritic cells (Im-dendritic cells) and lipopolysaccharide-treated DCs co-cultured with BMSCs for 48 h showed decreased expression of CD11c, CD80, CD86, IL-6, TNF-α and IFN-γ, and significantly increased expression of CD11b, IL-10, and TGF-β, stimulating splenocyte production of Treg markers (FOXP3, CD4, and CD25). MSCs induced the transformation of immature DC phenotype to regulatory dendritic cells (r-DCs), while MSCs secreting anti-inflammatory cytokines (IL-10 and TGF-β) played a similar role to r-DCs, leading to the activation of Treg [200]. MSCs regulated the differentiation and maturation of DCs, and the interaction between DCs and BMSCs upregulated IL-10 production by plasmacytoid dendritic cells (pDCs) and thus exerted anti-inflammatory effects [201]. MSCs inhibited the expression of toll-like receptor 3 (TLR3) and TLR9 and attenuated the migration of DCs by attenuating the expression of the lymph node-homing chemokine receptor CCR7 [202]. MSCs also inhibited dendritic cell maturation by downregulating DC maturation markers (MHC) major histocompatibility complex class II C, CD40, CD80, and CD86, directly impeding local antigen initiation by T cells [202,203]. In addition, secretion of IL-6, macrophage colony-stimulating factor (M-CSF), PGE2, and IL-10 in MSCs was associated with the induction of tolerogenic DCs [204].

MSCs inhibited myeloid DCs maturation, thereby preventing antigen presentation from DCs to T cells, and also inhibited tumor necrosis factor (TNF) to reduce the pro-inflammatory potential of DCs [205,206,207,208]. It also induced IL-10 production by pDCs to promote Treg production and inhibit immune system activation, thus contributing to the maintenance of homeostasis and tolerance to autoantigens in vivo [201,209].MSCs suppressed CD8+ cytotoxicity by inhibiting IFN-γ production and proliferation of CD4+ and CD8+ T lymphocytes, keeping T cells in the G0/G1 phase and supporting their survival in that state, and promoted CD4 + CD25+ Treg production by regulating immunosuppressive factors such as PGE2, IL-10, and diamino-2,3-dioxygenase (IDO) [189,210,211,212]. In this state, MSCs promoted the production of IL-4 and IL-10 by T cells and transformed T cells from a pro-inflammatory to an anti-inflammatory state, thus playing an important role in maintaining immune homeostasis [201,213,214]. MSCs directly induced regulatory T cell proliferation by releasing the immunomodulatory HLA-G isoform HLA-G5, which inhibited T cell proliferation and reduced NK cell and T cell cytotoxicity [215,216]. Through secretion of soluble trophic factors and immunomodulation, MSCs shifted the overall immunity from a pro-inflammatory Th1/Th17-driven immune response to a more anti-inflammatory Th2/Treg distribution, thereby halting the prolongation of the inflammatory phase and stimulating the progression of cell proliferation and tissue remodeling, which is essential for functional recovery of chronically injured organs [104]. In summary, MSCs intervene in T cell activation, proliferation, and phenotypic transformation and increase Tregs levels, thus exerting immunomodulatory effects. MSCs were transduced with GFP-HIF-1α lentiviral vectors (HIF-MSCs) and then co-cultured with DCs. HIF-MSCs were found to significantly decrease dendritic cell differentiation and increase resistance to NK cell lysis [217]. MSCs inhibited the cytotoxic activity of resting NK cells by downregulating the expression of NKp30 and natural-killer group 2, member D (NKG2D), inhibited NK proliferation, and inhibited IFNγ production [218]. MSCs interfered with NK cell degranulation and had an inhibitory effect on the proliferative killing activity of NK cells, which is associated with cytokines such as IL-2 and IFN-γ. IFNγ prompted MSCs to produce nitric oxide and indandiamine 2,3-dioxygenase (IDO), and IDO inhibited the production of IFN-γ through Tryptophan depletion, inhibited the proliferation of IFNγ-producing TH1 cells and blocked the activity of NK cells together with prostaglandin E2 (PG-E2) [219,220].

## 4. MSC-Derived Extracellular Vesicles

Exosomes (Exos) are a subset of intracellular multivesicular bodies (MVBs) that are generally thought to be 40–160 nm in size [221,222,223]. Endocytic vesicles form early sorting endosomes (ESEs) after encapsulating material inside and outside the cell. Some ESEs induce the formation of late sorting endosomes (LSEs), and the invagination of LSEs forms intraluminal vesicles (ILVs) after being encapsulated by MVBs, form exosomes via MVBs that have not been degraded by autophagosomes or lysosomes [224,225]. This process is carried out by endosomal sorting complex (ESCRT) proteins that sort the proteins they contain [226,227]. Exosomes carry thousands of cargoes, including proteins, lipids, and nucleic acids [228,229]. Proteins are always associated with membrane transport and fusion, and exosome identification. Lipids usually play a key role in exosome biogenesis, shape maintenance, and homeostatic regulation in recipient cells, exosomal lipids have some cell type specificity, and lipids play a key role in the formation and protection of exosome structures, vesicle biogenesis, and homeostatic regulation [230]. Nucleic acids are key regulators of intracellular communication and multiple signaling pathways [231,232,233]. Exosomes released from different parts of the same cell have different molecular compositions [234], and exosomes of different tissues and cellular origins carry different types of substances [235]. Microvesicles (EVs), also known as microparticles, exosomes, and membrane particles, share the same process of plasma membrane outgrowth or vesicle production as exosomes, except that Evs have a larger diameter, up to 1000 nm [221]. Their physiological properties are very similar to those of Exos, except that they carry more contents.

MSC-derived exosomes (MSC-Exos) and extracellular vesicles (MSC-Evs) serve as biological vesicles with a long circulating half-life, low immunogenicity, excellent permeability, and desirable biocompatibility. There is the expression of common surface biomarkers, such as CD81 and CD9, and MSC surface markers, such as CD29, CD44, CD73, and CD90 [236]. There is also the expression of endosomal markers CD63 and CD82 [9]. They share the same biological functions as MSCs, such as tissue regeneration, immune regulatory effects, and anti-inflammatory effects [237].

### 4.1. Bone Tissue

MSC-Exos and MSC-Evs played a significant role in promoting chondrocyte proliferation and migration, regulation of extracellular matrix anabolism, and promotion of vascular neogenesis. For example, synovial MSC-Exos had the ability to improve chondrocyte proliferation and migration both in vitro and in vivo [238]. Evs-rich angiotensin proteins promoted myeloid cell proliferation and extracellular matrix anabolism via the Notch1 signaling pathway. Sustained release of Evs ameliorates disc degeneration and promotes disc regeneration more effectively [239]. HIF-1α under hypoxic conditions impeded EFNA3 expression by inducing high miR-210-3p expression in MSC-derived small extracellular vesicles (MSC-sEVs), activated the PI3K/AKT pathway and promoted angiogenesis and bone regeneration [240]. Hypoxia-induced miR-17-5p enrichment in MSCs-sEVs, which in turn regulated the proliferation and synthesis of myeloid cell (NPC) stroma through the TLR4/PI3K/AKT pathway of the downstream target gene TLR4 for the treatment of intervertebral disc degeneration (IDD) [241]. Human umbilical cord mesenchymal stromal cell (MSC)-derived extracellular vesicles (hUCMSC-Evs) prevented bone loss and maintained bone strength in osteoporotic mice by enhancing bone formation, reducing bone marrow fat accumulation, and decreasing bone resorption. They are highly enriched in the potent bone-enabling protein CLEC11A (C-type lectin structural domain family 11, member A). Furthermore, in terms of differentiation regulation, hUCMSC-Evs enhanced the transition from lipogenic to osteogenic differentiation of bone marrow mesenchymal stem cells by delivering CLEC11A. The inhibitory effect of hUCMSC-Evs on osteoclast formation was also noted [242].

### 4.2. Liver and Kidney Tissue

The extracellular vesicles released by MSCs had significant hepatoprotective effects and promoted the proliferation and maturation of cells associated with liver function. For example, human Wharton’s jelly MSC-Exos (hWJ-MSC-Exos) limited liver failure (LF) and protected hepatocytes by inhibiting epithelial-tumor mesenchymal transition and inactivating the TGFβ1/SMAD2 pathway [243]. Choroidal plate-derived MSC-Exos carried miR-125b, which shows hepatoprotective effects by blocking Smo production and consequently inactivating the Hedgehog signaling pathway [244]. ADMSC-Exos delivered miR-122 to hepatic stellate cells (HSCs) and regulated miR-122 target genes IGF1R, Cyclin G1 (CCNG1), and proline-4-hydroxylase α1 (P4HA1) expression, thereby promoting cell proliferation and collagen maturation in hematopoietic stem cells [245]. In addition, MSC-Evs may also exert protective effects through the delivery of mitochondria. For example, hUCMSC-Evs treated hepatic ischemia-reperfusion injury (IRI) by transferring functional mitochondria to intrahepatic neutrophils and repairing their mitochondrial function in inhibiting the formation of neutrophil extracellular traps (NETs) in local liver tissue [246]. MSC-Evs inhibited Wnt/β-linked protein pathway components α-SMA and type I collagen expression, thereby preventing stellate cell activation and increasing hepatocyte regeneration. In vivo injection of hBMSC-Exos has been shown to be effective In alleviating ccl4-induced liver fibrosis and restoring liver function in rats [247]. MSC-eVs significantly reduced the mRNA expression of kwashiorkor cells (KCs) and inflammatory factors and activated the hepatic stellate cell (HSC) and LPS/TLR4 signaling pathways, thereby reducing inflammation and fibrosis [248]. Overexpression of MiRNA-181-5p in ADMSC-eVs downregulated transcription 3 (STAT3) and Bcl-2 in HST-T6 cells and activated autophagy, while type I collagen, waveform protein, a-SMA, and fibronectin were significantly reduced in the liver [249]. miR-122 overexpression regulated the expression of target genes such as insulin-like growth factor receptor 1 (IGF1R) cell cycle protein G (GNG1) and proline-4-hydroxylase P4HA11 (P4HA1) expression, resulting in more effective blockade of hematopoietic stem cell proliferation and collagen maturation [245]. miR-486-5p, which is highly expressed in MSC-eVs, targeted the hedgehog receptor and inhibited hedgehog signaling, thereby attenuating hematopoietic stem cell activation and liver fibrosis [250].

MSC-released extracellular vesicles effectively promoted renal endothelial cell proliferation and inhibited apoptosis. mRNAs encoding CDC6, CDK8, and CCNB1 from BMSC-Exos synergistically regulated renal tubular epithelial cell cycle entry and proliferation and blocked apoptosis [251]. ADMSC-Eoxs decreased pro-inflammatory cytokines, Smad3, and TGFβ fluorescent proteins by downregulating anti-apoptotic proteins, reducing creatinine and blood urea nitrogen (BUN) levels, and improving renal function [252]. hWJMSC-Exos inhibited NADPH oxidase (NOX) and reactive oxygen species from triggering Nrf2/antioxidant response elements, improved renal function, and inhibited apoptosis [253]. hUCMSC-eVs carried the mitochondria-located antioxidant enzyme MnSOD (manganese superoxide dismutase), which effectively reduced renal oxidative stress [254]. BMSC-eVs transferred anti-apoptotic mRNA to recipient cells and exerted pro-survival effects on renal cells in vivo and in vitro [255].

MSC-released extracellular vesicles also protected the kidney by over-promoting angiogenesis and cell migration. For example, ADMSC-Exos expressing glial cell line-derived neurotrophic factor (GDNF) reduced peritubular capillary thinning and renal failure, stimulated angiogenesis, cell migration, SIRT1 signaling pathway, and conferred apoptosis resistance [256]. BMSC-Exos was able to promote renal tubular epithelial cell proliferation by delivering IGF1 receptor mRNA [257]. MSC-eVs promoted renal tubular epithelial cell proliferation by inhibiting apoptosis in 70% of foot cells, promoting vascular regeneration and cell survival (increasing CD31+/Ki-67+ endothelial area) to prevent renal complications [258]. MSC-eVs reduce apoptosis and restore the expression of angiogenic factors in stenotic kidneys [259].

In the anti-inflammatory and anti-fibrotic context, MSC-eVs reduced the levels of several pro-inflammatory cytokines in renal veins, including TNF (tumor necrosis factor)-α and IL-6, while increasing the levels of IL-10. The number of reparative M2 macrophages in the renal parenchyma immunomodulatory effects were associated with increased expression of pro-angiogenic factors in the kidney after stenosis, restoring intrarenal microcirculation and improving medullary oxygenation and fibrosis 12% and thus glomerular filtration rate (GFR) in stenosed kidneys [260]. MSC-Exos ameliorated renal fibrosis by inhibiting YAP via casein kinase 1δ (CK1δ) and E3 ubiquitin ligase β-TRCP and blocking the RhoA/ROCK pathway by inhibiting the ROS-mediated p38MAPK/ERK signaling pathway [261,262,263]. MSC-Exos inhibited apoptosis and stimulated tubular epithelial cell proliferation, thereby reducing acute kidney injury, and thus protecting these kidneys from late CKD. MSC-eVs improved peritubular by reducing macrophage accumulation and fibrosis, inhibiting pro-inflammatory and pro-fibrotic proteins CX3CL1 [264]. MSC-eVs inhibited epithelial-mesenchymal transition capillary thinning and reduced tubular interstitial fibrosis to prevent the progression of renal injury [265]. Multiple injections of human liver stem cell-like cells (HLSCs) and MSC-eVs significantly improved renal fibrosis and type I collagen expression, with significant downregulation of related genes (Serpina1a, FAS ligand, CCL3, TIMP1, MMP3, type I collagen and SNAI1) and reduced DN symptoms [266]. In addition, it has been shown that various miRNAs carried by MSC-eVs play a role in renal protection. For example, miR-24, an important regulator of vascular inflammation, is involved in EV-induced post-ischemic renal repair [267,268]. miR-34c-5p inhibited the core focus (CF) of the cd81- EGFR complex, which ameliorated renal interstitial fibrosis (RIF) [269]. miRNA-215-5p inhibited ZEB2 and improved diabetic nephropathy (DN) symptoms [270,271]. miR-451a inhibited the expression of cell cycle inhibitors P15 and P19 by targeting their 3’-UTR sites, thereby decreasing α-SMA and increasing e-calmodulin expression. This resulted in EMT reversal and amelioration of DN symptoms [272].

### 4.3. Cardiovascular

Similar to MSCs, MSC-Exos and MSC-eVs protect cardiac function mainly by preserving cardiomyocytes, anti-apoptosis, reducing infarct area size, promoting endothelial cell proliferation, promoting revascularization, inhibiting fibrosis, inhibiting ECM remodeling, and modulating immunity. In terms of reducing infarct size, anti-apoptosis, and inhibiting ECM remodeling, MSC-Exos promoted hydrogen peroxide-induced proliferation and apoptosis of H9C2 cells and prevented TGF-β-induced conversion of fibroblasts to myofibroblasts [273]. The lncRNA KLF3-AS1 in BMSC-Exos inhibited H9C2 apoptosis and slowed down the progression of myocardial infarction via the lncRNA KLF3-AS1/miR138-5p/Sirt1 pathway [274]. Human-induced exosomes (hiPSC-MSC-Exos) from pluripotent stem cell-derived mesenchymal stem cells rapidly re-epithelialized, promoted collagen maturation and reduced scar size, promoted cell proliferation and migration, and increased type I and III collagen and elastin secretion in a dose-dependent manner [275]. Timp2-modified hUCMSC-Exos reduced TGF-β-induced secretion of MMP2, MMP9, and α-SMA in cardiac fibroblasts and inhibited ECM remodeling [276]. MSC-Eoxs exerted anti-fibrotic and infarct size reduction effects by delivering various miRNAs such as miR-19a, miR21, miR-22, miR-23a, miR-24, miR-29, miR -125b, miR-145, miR-221. miR-19a exerted anti-fibrotic and infarct size reduction effects by downregulating phosphatase and tensin homolog (PTEN) and triggering Akt and ERK signaling pathways [155]. miR21 inhibited PTEN, upregulated Akt, Bcl-2, and VEGF, promoted recovery of cardiac function, and reduced infarct size [125]. Dysregulation of miR-21-5p in exosomes of heart failure patients impaired regenerative activity, and restoration of miR-21-5p expression accelerated cardiac repair by enhancing cardiomyocyte and endothelial cell survival through phosphatase and tensin homolog/Akt pathways [277]. miR-22-enriched exosomes accelerated cardiac repair by targeting methyl-CpG-binding protein2 (MECP2), significantly reducing infarct size to decrease fibrosis [278]. miR-21, -23a, -125b, and -145 miRNAs in hWJMSC-Exos and hUCMSC-Eoxs hindered scar formation and myogenic cell accumulation by blocking and reducing collagen deposition through the TGFβ2/SMAD2 pathway [279]. MiR-29 and miR-2 inhibited TGF-β-induced fibrosis in fibroblasts, thereby enhancing cardiac repair [273]. miR-221 protected the heart by downregulating p53 and upregulating apoptosis regulator (PUMA) anti-apoptosis [280]. Cardiac miRNAs (miR-1, miR133a, miR-208a/b, and miR-499), which are extremely relevant to myogenesis, cardiac function, and pathology, have been detected in large numbers in the myocardium. These cardiac-specific miRNAs could greatly contribute to enhancing the regenerative properties and survival of stem cells [281].

MSC-Exos had the ability to promote recovery of cardiac function by promoting tubule generation from endothelial cells, inhibiting T cells, and reducing infarct size [282]. Its carriage containing encoded basic matricellular growth factor (bFGF), IGF1, and VEGF could be pro-angiogenic [154,283]. hWJMSC-Exos promoted wound healing and angiogenesis in vivo by transferring Wnt4 and activating β-linked protein [284]. miRNAs in MSC-Exos also have mostly pro-angiogenic effects. For example, miR-21 showed superior cardioprotective effects through the angiogenesis of the PTEN/Akt pathway [285]. miR-125a regulated endothelial tip cell generation by downregulating the expression of angiogenesis inhibitor delke4 (DLL4) [126]. miR-132 increased tube formation in HUVEC by targeting RASA1, enhanced neovascularization in the peri-infarct zone, and preserved cardiac function [286]. miR210 promoted angiogenesis and protected cardiac function both in vivo and in vitro. miR-210 also significantly improved angiogenesis by increasing the proliferation, migration, and tube formation capacity of HUVECs and contributed to the improvement of cardiac function after myocardial infarction [285]. miR-494 promoted muscle regeneration by improving angiogenesis and myogenesis [287]. miR-612 promoted muscle regeneration by miR-612-TP53-HIF-1α-VEGF axis, promoted paracrine hypoxia-inducible factor 1-α (HIF-1α)-VEGF signaling in HBMECs, which promoted the proliferation, migration and angiogenic activity of human brain microvascular endothelial cells (HBMECs) and promoted angiogenesis [288]. Many bioengineering tools were applied to exocytotic vesicles to enhance the efficacy, such as ADMSC-Exos overexpressed SIRT1 to restore cell migration and tube formation through the Nrf2/CXCL12/CXCR7 pathway, as well as the recruitment of EPCs to the repair region [289]. Macrophage migration inhibitory factor-engineered hUCMSC-Exos significantly enhanced proliferation, migration, and angiogenesis by delivering miR-133a-3p to HUVEC [290]. TIMP2-modified hUCMSC-Exos promoted the secretion level of Sfrp2, thereby promoting HUVEC proliferation, migration and tube formation, and angiogenesis [276]. Akt-modified PDGF-D in hUCMSC-Exos improved myocardial infarction treatment more effectively by promoting angiogenesis [122]. TIMP-2 modified hUCMSC-Exos increased angiogenesis by reducing cardiomyocyte apoptosis and enhanced cardioprotection by limiting ECM remodeling in part through activation of the Akt/Sfrp2 pathway [291]. Lentiviral CXCR4-transduced MSC-Exos significantly increased IGF-1α and pAkt levels, inhibited active cysteine 3 levels in cardiomyocytes, and promoted angiogenesis by enhancing VEGF expression, contributing to neovascularization and reducing infarct size and improving cardiac remodeling [154]. Packaging of long-stranded non-coding RNA H19 in BMSC-Exos regulated miR-675 expression, activated pro-angiogenic factor VEGF and intercellular adhesion molecule-1, and promoted endothelial cell proliferation exerting significant cardioprotective effects [292].

MSC-Exos ameliorated the myocardial inflammatory microenvironment in mice with dilated cardiomyopathy by significantly reducing M1 macrophages in the blood and heart and promoting macrophage conversion from the M1 phenotype to the M2 phenotype, compounding the reduction in plaque size and macrophage infiltration and promoting the restoration of cardiac function [293,294]. MSCs-Exos accumulated in mediastinal lymph nodes, and histocompatibility complex (MHC)-II antigen-presenting cells (APCs) uptake induced Foxo3 activation via the protein phosphatase (PP)-2A/p-Akt/forkhead box O3 (Foxo3) pathway, and Foxo3 induced APCs to express IL-10, IL-33, and IL-34 and other cytokines and established Treg-induced ecotone in MLN, with enhanced Treg differentiation and cardiac targeting, thereby promoting cardiac inflammation regression and cardiac repair after MI [295]. Exosomes obtained from LPS-pretreated BMSCs increased M2 macrophage polarization and attenuated post-myocardial infarction inflammation by inhibiting the LPS-dependent NF-κB signaling pathway and activating the AKT1/AKT2 signaling pathway [296]. MSC-Exos affected DCs, monocytes, and macrophages, constructed an anti-inflammatory environment, enhanced Treg polarization, and through downstream c-Fos proteins, significantly improved ischemia-reperfused cardiac function and impaired perfusion in infarcted mice [297]. MSC-Exos carried many miRNAs involved in suppressing the inflammatory response to exert cardioprotective effects. For example, miR-34a, miR-124, and miR-135b [298], miR-125b mediated the p53-BNIP3 signaling pathway [299], miR-25-3p targeted the pro-apoptotic genes FASL and PTEN [300], miR-146a interacted with the 3’-untranslated region of EGR1 [301], miR-221/222 interacted with the 3’-untranslated region of EGR1 via PUMA/ETS-1 [302], miRNA-181a down-regulated the pro-inflammatory cytokine TNF-α and IL-6, increased the anti-inflammatory cytokine IL-10 and promoted Treg cell polarization [303], miR-10a promoted Th17 and Treg responses [304], micriRNA-133 inhibited Snail1 inhibition [305], and miR-146 down-regulated early growth response factor 1 (EGR1) [301].

### 4.4. Neuro

MSC-Exos restored neurological damage after ischemia, inhibited inflammation-induced neurodegeneration microglia proliferation, suppressed reactive astrocyte proliferation, promoted angiogenesis and neuronal growth rate after brain injury, increased vascular density and generation, increased the number of new neuroblasts, reduced inflammation, improved functional recovery, and provided long-term neuroprotection [252,306,307,308]. MSC-Exos inhibited the expression of pro-apoptotic Bcl-2-associated X protein, TNF-α, and IL-1β upregulated the anti-apoptotic protein B-cell lymphoma 2 and regulated microglia/macrophage polarization to reduce injury size and restore neurobehavioral performance [309]. Intravenous administration of BMSC-Exos exhibited an anti-inflammatory response in injured spinal cord tissue and improved motor activity via astrocytes and microglia [310]. ADMSCs-Exos contained a large amount of neurotolerin, which degraded β-amyloid peptides and reduced both secreted and intracellular β-amyloid peptides in neuroblastoma cells [311]. MSC-Exos in the Alzheimer’s disease (AD) APP/PS1 mouse model rescued synaptic dysfunction and promoted anti-inflammatory effects [312]. MSC-Exos carried multiple miRNAs such as miR-17-92 cluster, miR-133b, and miR-322. miR-133b promoted neurosynaptic growth by downregulating RhoA expression, contributing to neurosynaptic remodeling and stroke recovery [313]. miR-17-92 cluster mediated neurogenesis, neural remodeling, and oligodendrocyte generation in the ischemic border zone. miR- 17-92 cluster-enriched Exos repressed PTEN, the binding target gene of miR-17-92 cluster, activating downstream proteins, protein kinase B (the mechanistic target of rapamycin), and glycogen synthase kinase 3β [314]. miR-322 repressed the expression of pknox1, a protein that inhibits the M1 phenotype of macrophages, thereby promoted the M2 phenotype transformation [188].

MSC-eVs reduced brain injuries and infiltration of other types of immune cells, such as monocytes/macrophages and lymphocytes, creating an environment conducive to neuronal recovery [187,315,316,317]. MSC-eVs increased endothelial cell proliferation and reduced pro-inflammatory astrocyte and microglial cell activation [308,318,319]. Activated microglia, in turn, promoted the enrichment of miRNAs by MSCs-eVs, and enhanced immunomodulatory potential against neuroinflammation [320]. MSC-eVs also contained angiopoietin 1, Notch 2, vascular cell adhesion molecule 1 (VCAM-1), and transforming growth factor-β2 (TGF-β2). These molecules promoted survival and neuroprotection and promoted angiogenesis in damaged tissues [260]. MSCs-EVs also contained ANXA1, which exerted stabilizing and anti-inflammatory effects on the endothelium through the ANXA1/FPR axis [321,322]. MSC-EVs reduced the number of beta-amyloid (Aβ) plaques and slowed down the pathogenesis of AD [323]. MSC-EVs exerted protective effects against hypoxic-ischemic (HI) injury-induced neurological damage through the delivery of miR-21a-5p [324]. MSC-EVs containing miR-182 negatively regulated TLR4/NF-κB pathway in macrophages and promoted M2 polarization [293]. MSC-EVs delivered miR-21a to microglia, induced anti-inflammatory M2 polarization, and enhanced neuroprotection [324]. MSC-EVs containing miR-184 have also been shown to promote neurogenesis [325].

### 4.5. Immunomodulation

MSC-Exos is involved in immune regulation by regulating the function of immune cells and altering the secretion of inflammatory factors such as TNF-α and IL-1β [326]. MSC-Exos significantly reduced the inflammatory response by upregulating IL10 and downregulating the expression of TNF-α and IL6 [327]. MSC-Exos also exerted its anti-inflammatory effects by increasing IL10 levels, and decreasing pro-inflammatory such as IL-1β and TNF-α mRNA levels of cytokines exerted anti-inflammatory effects [328]. Hypoxia increased miR-205-5p levels in BMSC-Exos and effectively suppressed inflammation through the miR-205-5p/PTEN/AKT pathway [329]. miR-21-5p in gingival mesenchymal stem cell (GMSC) exosomes played a key role in neuroprotection and anti-inflammation by binding to programmed cell death 4 (PDCD4) in the TG exon [330]. MSC-Exos enhanced IL-1β-induced cell proliferation and inhibited apoptosis and inflammation [331]. MSC-Exos inhibited NLRP3 inflammatory vesicle components (NLRP3, caspase1-p20, ASC) and gasdermin D (GSDMD-F, GSDMD-N) in BV2 microglia via miR-146a-5p/TRAF6, attenuated inflammatory response and increased autophagy levels to inhibit apoptosis [332]. MSCs-sEVs carried substances that change with the secretory cell state when MSCs are in an inflammatory environment. They released more functional growth factors, exosomes, and chemokines. Both inflammation-stimulated ADMSC-sEVs (IAE) and normal ADSC-sEVs (AE) promoted cell proliferation; IAE also significantly improved cell migration, and high expression levels of miR-27b-3p in IAE-regulated macrophages by targeting macrophage colony-stimulating factor-1 (CSF-1), significantly promoting M2 macrophage differentiation [333]. High tyrosine phosphatase-2 (SHP2)-expressing MSC-EVs-SHP2 with high blood–brain barrier permeability effectively delivered SHP2 to the brain of AD mice, significantly induced mitochondrial phagocytosis of neuronal cells, and attenuated mitochondrial damage-mediated apoptosis and NLRP3 inflammatory vesicle activation [334]. BMSC-EVs reduced the expression of the pro-inflammatory cytokines IL-1β, TNF-α, and IL-6, and enhanced the expression of IL-10, chondrogenic genes, type II collagen, and SOX9 [335]. BMSC-EVs also significantly ameliorated inflammation in vivo by upregulating miR- 34a-targeted inhibition of the cell cycle protein 1-activated ATM/ATR/p53 signaling pathway, thereby inhibiting RA fibroblast-like synoviocytes (RA-FLSs) [336].

MSC-Exos promoted the differentiation of monocyte myeloid-derived suppressor cells (M-MDSC) into highly immunosuppressive M2-polarized macrophages [337]. ADMSC-Exos transferred stem cell-derived mitochondrial components to alveolar macrophages, increased mtDNA levels, MMPs, OXPHOS activity, and ATP production, downregulated IL-1β, TNF-α and iNOS secretion, increased production of anti-inflammatory cytokines IL-10 and Arg-1, and improved macrophage mitochondrial integrity and oxidative phosphorylation levels, thereby restoring metabolic and immune homeostasis of airway macrophages and reducing lung inflammatory pathology [338].

MSC-Exos decreased serum alanine aminotransferase (ALT) and pro-inflammatory cytokine levels while increasing the expression of anti-inflammatory cytokines, downregulating pro-inflammatory factors, and inducing Treg activity [339,340]. MSCs-Exos accumulated in mediastinal lymph nodes, induced Foxo3 activation, promoted the expression of cytokines such as IL-10, IL-33, and IL-34 by APCs, and promoted the differentiation of Treg for inflammation [295]. MSCs-Exos inhibited the maturation of LPS-treated DCs and thus attenuated antigen presentation [341]. ADMSC-Exos improved type 1 autoimmune diabetes mellitus (T1DM) symptoms by upregulating the expression of regulatory T cells, interleukin 4 (IL 4), IL 10, and transforming growth factor- (TGF-β) and downregulating IL-17 and IFN-γ [342]. hBMSC-EVs increased PRG4, BCL2 and ACAN gene expression and reduced apoptosis by downregulating MMP13, ALPL, IL-1β-activated pro-inflammatory Erk1/2, PI3K/Akt, p38, TAK1, NF-K1 and NF-κB signaling pathways [343].

## 5. Cytokines

Growth factors like HGF, IGF-1, VEGF, FGF2, EGF, PDGF, SDF-1, keratinocyte growth factor, angiopoietin-1, erythropoietin and thrombopoietin released from MSCs played important roles in promoting cell proliferation and tissue damage repair [95,96,97,98,344]. MSCs secreted anti-apoptotic and anti-inflammatory factors IL-6, IL-1Ra, IL-10, and TGF-β, which play immunomodulatory roles such as anti-fibrosis, inhibiting pro-inflammatory cell activation and promoting Treg [108,182,200]. MSCs affected the cytokine secretion of other cells. For example, by regulating the polarization of macrophages, MSCs affect the level of cytokines secreted by macrophages such as TNF-α, IL-1β, IL-1α, IL-6, IL-12, TGF-β, IL-10, HGF, PGE2, etc., further exerting anti-inflammatory and other effects [119,136,137,188]. MSCs also secrete enzymes to participate in metabolic regulation, such as producing SOD3 to reduce ROS levels [135].

MSC-Eoxs and MSC-EVs also decreased tissue levels of pro-inflammatory factors TNF-α, IL-6, HMGB1, decreased oxidative stress factors NOX1, NOX2, and increased levels of anti-inflammatory factors IL-10, Arg-1, promoted Treg activation, regulated IL-33, IL-34 level [252,260,295,338,345]. MSCs and MSC-Eoxs together had better reduced TNF-α, NF-κB, IL-1β, macrophage migration inhibitory factor, plasminogen activator inhibitor, COX-2 inflammatory factors, reduced Smad3, TGF-β, increased Smad1/5, BMP-2 and inhibited fibrosis in tissues by downregulating apoptotic proteins [252].

Cytokines also have an effect on MSCs. For example, IFN-γ and IL-1β increased the level of VEGF secreted by MSCs [149]. And IFN-γ promoted the production of IDO by MSCs, which in turn inhibited IFN-γ production, TH1 cell proliferation, and NK cell activity [219,220]. Adipokines are a kind of adipocyte-derived factor with immunomodulatory properties. Visfatin induced the secretion of IL-6, IL-8, and MCP-1 during osteogenic and adipogenic differentiation. In contrast to resistin and leptin, visfatin increased MMP2 and MMP13 during adipogenesis. In osteogenically differentiated cells, MMPs and TIMPs were reduced by visfatin. Visfatin significantly increased matrix mineralization during osteogenesis, whereas collagen type I expression was reduced [346]. FGF2 and EGF increased MSCs proliferation, while TGF-β1 decreased MSC proliferation. FGF2, EGF and TGF-β1 increased MSCs cytokine levels, such as IL-6, CXCL8, CCL2, interferon-gamma and TNF-α, and decreased CCL5, PDGF-BB, and IL-10 levels [347]. Note that MSCs of different origins may have different cytokine releases and have different effects on other cellular tissues [348].

## 6. Summary and Prospect

**Figure 1:**
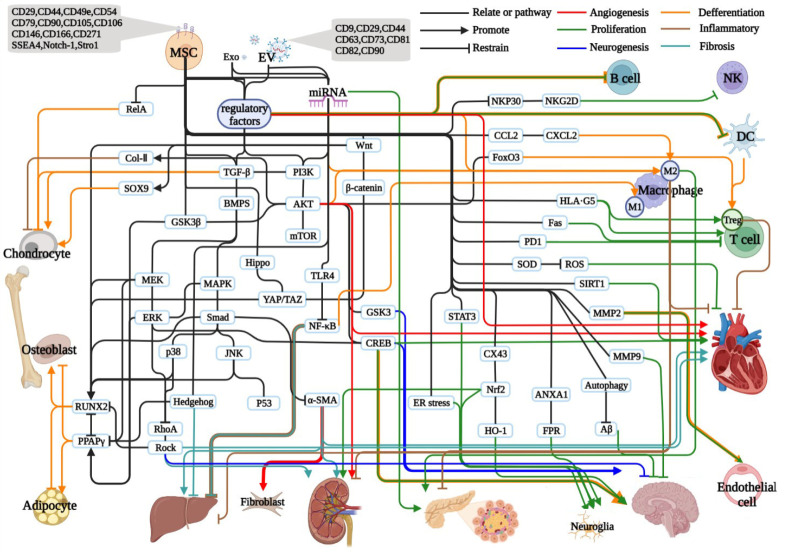
Effects and regulation of MSCs and their exocytotic vesicles in different systems. All solid black lines indicate association or signaling pathways at both ends, solid lines of each color with arrows indicate a promoting relationship, solid lines of each color with horizontal bars represent an inhibiting relationship, solid red lines represent angiogenesis promoting (arrows) or inhibiting (horizontal bars) relationships, solid green lines represent proliferation or apoptosis relationships, blue represents neurogenesis, neuronal proliferation or related inhibition, orange represents promotion or inhibition of differentiation, brown represents promotion or inhibition of inflammatory responses, and cyan represents promotion or inhibition of fibrosis.

In summary, MSCs and their exocytotic vesicles have been widely noticed and applied for their various roles in cell preservation, proliferation, migration, anti-inflammation, anti-apoptosis, anti-fibrosis, angiogenesis, neurogenesis, metabolism, immunomodulation (Figure 1). However, it is undeniable that MSCs have conflicting manifestations in some studies, such as whether BMSCs inhibited B cell proliferation [140,189,190,195]. Perhaps a deeper and more detailed study of MSCs to confirm the different characteristics of MSCs from different sources at different stages of culture and transplantation may help us to better understand MSCs. At present, a research team has sequenced single-cell RNA of MSCs, obtained the development trajectories of five subpopulations, and revealed the differentiation path from the stem-like proliferating cells (APC) to pluripotent progenitor cells, which can help define and classify MSCs.

The exocytotic vesicle inclusions of MSCs vary depending on the MSCs and their status. There are no strict and valid criteria for their acquisition, resulting in significant differences and uncertainties in their effects. For example, BMSC-EVs appeared to have a greater angiogenic potential than ADMSC-EVs, with an approximately 4-fold increase in the number of endothelial cells in the former compared to the latter [318]. EVs from endometrial MSCs produced higher levels of angiogenesis than came from BMSC-EVs or BMSC-EVs [125]. Treatment with BMSC-EVs increased bone volume 4-fold compared to controls [349], whereas ADMSC-EVs increased bone volume approximately 1.33-fold [350]. Both BMSC-EVs and ADSC-EVs induced macrophage M2 polarization in vivo and in vitro in acute lung injury mouse model, BMSC-EVs resulted in a significant increase (3.2-fold) in the expression of the m2-polarization marker CD206 [351]. In contrast, another study showed that ADMSC-EVs only increased by 1.5-fold in M2-polarization capacity [352]. This may require more studies on the exocytotic vesicles of different MSCs and the development of efficient extraction identification criteria. One study has already proposed a protocol for extracting six different subpopulations of extracellular vesicles from tissues [353]. Furthermore, hUCMSC-Exos promoted the initiation of dormancy in breast cancer and conferred resistance to conventional chemotherapy, and tumor dormancy is closely associated with tumor recurrence, metastasis, and chemoresistance [354,355], which is not the result we had hoped for. Due to the multi-substance-carrying capacity of exosomes and the resulting multidirectional potential, we need to purify their carriers to remove relatively harmful substances in specific therapeutic regimens to ensure therapeutic efficiency and safety [356]. Finally, the targeting of MSCs exocytotic vesicles is also of our concern. After intravenous injection, which would be the choice in most studies, vesicles are significantly taken up mainly by relevant ductal epithelial cells, peripapillary endothelial cells, and macrophages, thus exploring effective targeting techniques could help to improve the efficacy, such as combining autohistochemical peptides to enrich for relevant targeting molecules [119,357], enriching relevant target molecules [259]. Therefore, combining bioengineering may help us to obtain more desirable efficacy.

## Data Availability

The data that support the findings of this study are available from the corresponding author upon reasonable request.
